# Electrochemical Oxidation of Resorcinol in Aqueous Medium Using Boron-Doped Diamond Anode: Reaction Kinetics and Process Optimization with Response Surface Methodology

**DOI:** 10.3389/fchem.2017.00075

**Published:** 2017-10-13

**Authors:** Bahadır K. Körbahti, Pelin Demirbüken

**Affiliations:** Faculty of Engineering, Chemical Engineering Department, Mersin University, Mersin, Turkey

**Keywords:** boron-doped diamond, electrochemical oxidation, optimization, reaction kinetics, resorcinol, response surface methodology, wastewater treatment

## Abstract

Electrochemical oxidation of resorcinol in aqueous medium using boron-doped diamond anode (BDD) was investigated in a batch electrochemical reactor in the presence of Na_2_SO_4_ supporting electrolyte. The effect of process parameters such as resorcinol concentration (100–500 g/L), current density (2–10 mA/cm^2^), Na_2_SO_4_ concentration (0–20 g/L), and reaction temperature (25–45°C) was analyzed on electrochemical oxidation using response surface methodology (RSM). The optimum operating conditions were determined as 300 mg/L resorcinol concentration, 8 mA/cm^2^ current density, 12 g/L Na_2_SO_4_ concentration, and 34°C reaction temperature. One hundred percent of resorcinol removal and 89% COD removal were obtained in 120 min reaction time at response surface optimized conditions. These results confirmed that the electrochemical mineralization of resorcinol was successfully accomplished using BDD anode depending on the process conditions, however the formation of intermediates and by-products were further oxidized at much lower rate. The reaction kinetics were evaluated at optimum conditions and the reaction order of electrochemical oxidation of resorcinol in aqueous medium using BDD anode was determined as 1 based on COD concentration with the activation energy of 5.32 kJ/mol that was supported a diffusion-controlled reaction.

## Introduction

Resorcinol (1,3-dihydroxybenzene, C_6_H_6_O_2_, CAS No. 108-46-3) is a white crystalline phenolic compound and it is a main component of the adhesives in rubber products and wood additives. The other uses include the manufacture of specialty chemicals, explosive primers, antioxidants, flame retardants, UV stabilizers, dyes, agricultural chemicals, fungicidal creams and lotions, meta-amino phenols, hair dyes, and pharmaceuticals (Hahn et al., 2006). Resorcinol can be released into the environment as hazardous pollutant from industrial production, coal conversion processes, oil shale mining refineries, and consumer uses such as hair dyes and pharmaceuticals. Although in low tonnage, resorcinol is most common in hair dyes, shampoos, hair lotions, peels, and in products used to treat acne, eczema, and other dermatological issues (Hahn et al., 2006). Therefore, resorcinol can be released into the environment during the production and processing of industrial facilities and from domestic effluents as well. The exposure to resorcinol causes toxicological effects in humans and animals which were reported as thyroid dysfunction, skin irritation, central nervous system disturbances, red blood cell changes, and decreases in body weight gain and decreased survival (Hahn et al., 2006).

The scientific studies demonstrate that resorcinol has both *in vivo* and *in vitro* antithyroid activity (Lynch et al., 2002). Resorcinol inhibits the activity of thyroid peroxidase enzymes responsible for the incorporation of iodine into tyrosine residues during the synthesis of thyroid hormone. Clinical case reports documented that continuous exposure to high doses of resorcinol with large amounts of resorcinol containing ointments over a long period of time for months to years may induce reversible hypothyroidism (Lynch et al., 2002). It was reported that thyroid effects may occur as a result of dermal exposure to integrity-compromised skin at resorcinol dose levels in the range 34–122 mg/kg/day based on the human data (Lynch et al., 2002).

The subchronic toxicity of resorcinol was evaluated in 17-day and 13-week studies in F344 rats and in B6C3F1 mice to establish the dose ranges for a 2-year carcinogenicity study (National Toxicology Program, 1992). National Toxicology Program (1992) reported that resorcinol at high doses to rodents can disrupt thyroid hormone synthesis and can produce goitrogenic effects. These effects were not seen in a 2-year bioassay at doses of up to 520 mg/kg/day (National Toxicology Program, 1992). Skowroñ and Zapór (2004) investigated the cytotoxicity in 3T3 fibroblast cells in short-term (3 h) and long-term (72 h, 6 weeks) exposure to resorcinol in the bioreactor. The authors reported that after 3 h of exposure the resorcinol caused inhibition of mitochondrial activity in concentrations of 2,500–4,000 μg/cm^3^ and inhibition of the intake of neutral red dye by lysosomes in concentrations of 1,500–4,000 μg/cm^3^ (Skowroñ and Zapór, 2004). Resorcinol in concentrations above 1 μg/cm^3^ caused inhibition of 3T3 fibroblast cell growth in the mitochondrial function and total cell protein tests after 72 h of exposure (Skowroñ and Zapór, 2004).

The environmental exposures of the general human population to resorcinol may be the result of drinking water consumption and through air inhalation. Resorcinol may be present in ground water at trace to low levels especially in areas where aquifers flow through rocks that are rich in organic material (Lynch et al., 2002). However, there is a lack of information in the literature from which source to quantify the environmental exposures of the general human population to resorcinol through drinking water and the inhalation of ambient air (Lynch et al., 2002).

In the literature, several biological, physical, chemical, physicochemical, and advanced oxidation processes have been investigated for the abatement of phenolic compounds in wastewater due to their hazardous and toxicological effects (Zareie et al., 2001; Körbahti et al., 2002; Cañizares et al., 2005b, 2006; Nasr et al., 2005; Duan et al., 2013; Li et al., 2013). Electrochemical processes are the most promising technology that offers competitive advantages, effective oxidation performance and environmental compatibility in treatment of bio-refractory, toxic, and highly concentrated organic wastewater (Cañizares et al., 2005b; Comninellis and Chen, 2010; Sirés and Brillas, 2012; Duan et al., 2013; Li et al., 2013; Körbahti and Taşyürek, 2015; Jarrah and Mu'azu, 2016; Mu'azu et al., 2016).

In electrochemical processes, organic pollutants in aqueous medium can be removed by direct and indirect mechanisms depending on the properties of the anodes and process conditions (Comninellis and Chen, 2010; Jin et al., 2014). Direct oxidation occurs at the anode surface and indirect oxidation occurs in the aqueous medium by the production of redox reagents (Panizza and Cerisola, 2009; Comninellis and Chen, 2010; Brillas and Martínez-Huitle, 2015).

Boron-doped diamond (BDD) thin film is a new electrode material superior to TiO_2_, Pt, PbO_2_, IrO_2_, SnO_2_, and glassy carbon anodes due to its corrosion resistance, good conductivity, inert surface, mechanical and chemical stability, and increased mineralization rates at high current efficiencies (Chen et al., 2004; Nasr et al., 2005; Panizza and Cerisola, 2007; Comninellis et al., 2008; Weiss et al., 2008; Liu et al., 2009). Diamond films are not conductive, therefore doping with boron atoms make the diamond films electrical conducting. BDD thin films usually prepared on silicon, niobium, titanium, tungsten, molybdenum, tantalum, or glassy carbon materials by chemical vapor deposition (CVD) and the doping level of boron in the diamond layer can be expressed as B/C ratio which is about 1,000–10,000 ppm (Panizza and Cerisola, 2009).

In the literature, BDD anodes were investigated in electrochemical oxidation of organic pollutants such as carboxylic acids, pesticides, cyanides, pharmaceuticals, chlorobenzene, phenols and phenol derivatives, surfactants, textile dyes, and real wastewaters (Liu et al., 2009; Panizza and Cerisola, 2009; Sirés and Brillas, 2012; Brillas and Martínez-Huitle, 2015). During the electrolysis physisorbed hydroxyl radicals produce at BDD anodes by the oxidation of water molecules. Hydroxyl radicals assist the nonselective oxidation of organic compounds (R) and their reaction intermediates into CO_2_ and H_2_O which may result in complete mineralization (Panizza and Cerisola, 2009; Comninellis and Chen, 2010).

(1) $$BDD+H_{2}O\to BDD{(}^{•}OH)+H^{+}+e^{-}$$

(2) $$BDD{(}^{•}OH)\;+\;R\;\to \;BDD+CO_{2}+H_{2}O$$

Besides hydroxyl radicals produced by water oxidation, it is known that peroxodisulfate (S_2_${\text{O}}_{8}^{2-}$) oxidant also generates in sulfate medium at BDD anodes (Serrano et al., 2002; Cañizares et al., 2005a; Li et al., 2010; Sirés and Brillas, 2012; Davis et al., 2014). Once, Na_2_SO_4_ dissolves in the aqueous medium it ionizes into Na^+^ and ${\text{SO}}_{4}^{2-}$. Then peroxodisulfate (S_2_${\text{O}}_{8}^{2-}$) produces at high overpotential supplied by the BDD anode in the presence of ${\text{SO}}_{4}^{2-}$ ions in the electrolyte.

(3) $$Na_{2}SO_{4}\;\to \;2Na^{+}\;+\;SO_{4}^{2-}$$

(4) $$2SO_{4}^{2-}\;\to \;S_{2}O_{8}^{2-}+2e^{-}$$

The aim of this study was to investigate the electrochemical oxidation of resorcinol in aqueous medium using boron-doped diamond (BDD) anode in the presence of Na_2_SO_4_ supporting electrolyte. The effect of process parameters on COD removal, mass transfer coefficient (k_m_), ${\text{J}/\text{J}}_{\text{lim}}^{{}^{\circ}}$ values (α), and energy consumption was determined. The process optimization was accomplished using response surface methodology (RSM) and the reaction kinetics of electrochemical oxidation of resorcinol in aqueous medium using BDD anode were evaluated at response surface optimized conditions.

## Materials and methods

### Chemicals and materials

Resorcinol (Merck), Na_2_SO_4_ (Riedel-de Haën), HgSO_4_ (Merck), methanol (Merck), and Merck Spectroquant® 14541 COD cell tests were purchased in extra pure grade and used without purification. Double distilled water was produced using water still (GFL-2008) and ultrapure water system (Millipore Simplicity® UV) with the resistivity of 18.2 MΩ.cm@25°C, TOC < 5 ppb.

### Experimental set-up and procedure

DURAN® batch electrochemical reactor (Rettberg, Germany) with heating/cooling jacket used in this study. The net reaction volume was 600 mL. Three plates DIACHEM® boron-doped diamond (Nb/BDD) anode (CONDIAS, Germany) and 4 cylindrical (ϕ = 12.0 mm) iron cathode were used as electrodes with 15 mm anode/cathode spacing. Iron electrodes were purchased from local sources. Total electrode surface area was 280 cm^2^. Batch electrochemical reactor was equipped with programmable Goodwill PST-3201 power supply, Heidolph RZR 2021 mechanical mixer, thermometer, Memmert WB 22 heating/cooling water bath and Heidolph PD 5206 peristaltic pump. Reaction medium was mixed at 750 rpm. Samples were withdrawn from the electrochemical reactor at regular periods for the analytical measurements.

### Analytical measurements

HPLC analysis of resorcinol were conducted using Inertsil ODS-3 (5 μm, 4.6 × 250 mm) column in a Shimadzu Prominence LC-20AD Liquid Chromatography equipped with DGU-20A5 degasser, CBM-20Alite System Controller, LC-20AD gradient pump, SIL-20A auto sampler, CTO-20A column oven, and SPD-20A UV/Vis detector. Mobile phase consisted of methanol and water (75/25) at a flow rate of 1.0 mL/min. UV detection was performed at 270 nm wavelength. The column temperature and injection volume were set as 40°C and 30 μL, respectively. The regression coefficient of the calibration curve for resorcinol was *R*^2^ = 0.9991. pH was measured using WTW inoLab BNC720 model pH meter/conductivity meter. Merck Spectroquant® 14541 COD cell tests and Nova 60 water/wastewater photometer were used for the COD analysis.

### Design of experiments and process optimization using response surface methodology

Response surface methodology (RSM) is a mathematical and statistical method used in designing experiments, building models, evaluating the effect of process variables, and searching optimum operating conditions for the responses (Montgomery, 2009; Myers et al., 2009). The responses can be related to the factors by linear or quadratic models in RSM. The quadratic model, which also includes the linear model, is given in Equation (5) (Montgomery, 2009; Myers et al., 2009):

(5) $$y=\;\beta_{o}+\sum_{i=1}^{k}\beta_{i}x_{i}+\sum_{i=1}^{k}\beta_{ii}x_{i}^{2}+\underset{i}{\sum }\sum_{<j=2}^{k}\beta_{ij}x_{i}x_{j}$$

RSM is a very powerful tool that provides detailed mathematical and statistical analysis of the experimental results. In the literature, RSM was applied for the experimental design and process optimization in electrochemical oxidation of various types of wastewater containing textile dyes, pharmaceuticals, paint, dairy effluents, pulp and paper, phenols, phenol derivatives, landfill leachate, organic acids, and pesticides in most common using Box-Behnken design (BBD) and central composite design (CCD) (Nair et al., 2014). In this study, RSM was utilized using central composite design (CCD) for the electrochemical oxidation of resorcinol in aqueous medium at BDD anode. Central composite design (CCD) is the most popular class of second-order designs that provides very good predictions in the middle of the design space. CCD with four parameters at five levels was coded between the ranges of −2 and +2 using Design-Expert^©^ 10 as presented in Table 1. The runs were augmented in one block with 16 factorial, 8 axial, 6 center, and additional 8 axial points for the design space and carried out in randomized order in order to estimate pure error for the lack of fit test as shown in Table 2. The process variables (factors) were resorcinol concentration (100–500 mg/L), current density (2–10 mA/cm^2^), Na_2_SO_4_ electrolyte concentration (0–20 g/L), and reaction temperature (25–45°C); and the responses were chemical oxygen demand (COD) removal, mass transfer coefficient (k_m_), ${\text{J}/\text{J}}_{\text{lim}}^{{}^{\circ}}$ values (α), and energy consumption.

**Table 1:** Experimental design for the electrochemical oxidation of resorcinol in aqueous medium using BDD anode.

**Process parameters**		**Coded** α **levels**				
		**−2**	**−1**	**0**	**+1**	**+2**
***x***_1_	Resorcinol concentration (mg/L)	100	200	300	400	500
***x***_2_	Current density (mA/cm^2^)	2	4	6	8	10
***x***_3_	Na_2_SO_4_ concentration (g/L)	0	5	10	15	20
***x***_4_	Reaction temperature (°C)	25	30	35	40	45

**Table 2:** Central composite design (CCD) and experimental results for the electrochemical oxidation of resorcinol in aqueous medium using BDD anode.

**Run**	***x*_1_**	***x*_2_**	***x*_3_**	***x*_4_**	**V_m_**	**COD_o_**	**COD_f_**
					**(V)**	**(mg/L)**	**(mg/L)**
1	0	0	0	0	8.9	637	83
2	1	−1	−1	1	8.5	837	358
3	0	0	0	2	8.6	639	179
4	1	−1	1	−1	5.9	880	311
5	0	0	0	−2	11.4	665	155
6	−1	−1	1	1	5.6	444	95
7	2	0	0	0	8.5	1,068	167
8	−1	−1	−1	−1	8.3	445	147
9	−1	1	−1	−1	13.9	461	119
10	0	0	0	0	8.4	607	68
11	−1	1	−1	1	13.3	433	51
12	1	1	1	1	8.2	831	216
13	0	0	0	0	7.8	658	85
14	1	1	−1	−1	14.8	811	208
15	−1	1	1	1	7.3	434	74
16	−1	1	1	−1	8.0	419	55
17	1	−1	−1	−1	10.8	891	259
18	0	0	0	0	9.1	596	58
19	0	−2	0	0	6.5	602	352
20	0	0	0	0	7.7	632	73
21	0	0	2	0	7.2	631	93
22	−1	−1	−1	1	8.2	425	90
23	1	−1	1	1	5.9	825	266
24	0	0	−2	0	32.0	690	617
25	1	1	−1	1	15.0	788	101
26	−2	0	0	0	8.3	252	52
27	0	0	0	0	7.9	600	256
28	−1	−1	1	−1	6.5	438	113
29	1	1	1	−1	9.5	813	116
30	0	2	0	0	12.0	639	99
31	−1	0	0	0	8.2	440	64
32	1	0	0	0	9.7	840	82
33	0	−1	0	0	6.4	628	193
34	0	1	0	0	8.9	625	70
35	0	0	−1	0	12.0	634	256
36	0	0	1	0	7.2	644	117
37	0	0	0	−1	7.4	695	120
38	0	0	0	1	7.9	705	157

## Results and discussion

### Effect of process parameters

The effect of resorcinol concentration, current density, Na_2_SO_4_ electrolyte concentration and reaction temperature were investigated on electrochemical oxidation of resorcinol in aqueous medium using BDD anode in a batch electrochemical reactor. Mean cell voltage, initial and final COD values during the electrochemical treatment can be seen in Table 2. In the study, calculations were made by using the experimental results in Table 2 and analyzed for the process optimization using RSM. COD removal, mass transfer coefficient (k_m_), ${\text{J}/\text{J}}_{\text{lim}}^{{}^{\circ}}$ values (α), and energy consumption data were processed for Equation (5) including ANOVA in order to obtain the interaction between the process variables and the responses. The quadratic response surface models were obtained for the responses in Equations (6–9) and the statistical significance verified by the analysis of variance (ANOVA) in Table 3. In Equations (6–9), *y*_COD_, *y*_km_, *y*_α_, and *y*_EC_ are COD removal, mass transfer coefficient, ${\text{J}/\text{J}}_{\text{lim}}^{{}^{\circ}}$ values, and energy consumption, respectively; and *x*_1_, *x*_2_, *x*_3_, and *x*_4_ are process variables (factors) of resorcinol concentration (mg/L), current density (mA/cm^2^), Na_2_SO_4_ electrolyte concentration (g/L), and reaction temperature (°C), respectively.

(6) $$\begin{array}{l} y_{\text{COD}}=0.010955x_{1}+10.55437x_{2}+10.92951x_{3}+3.72522x_{4}+8.46875\times 10^{-3}x_{1}x_{2}\\ \;-\;1.48750\times 10^{-3}x_{1}x_{3}-4.46250\times 10^{-3}x_{1}x_{4}-0.036875x_{2}x_{3}+0.034375x_{2}x_{4}\\ \;-\;0.082750x_{3}x_{4}+1.60235\times 10^{-4}x_{1}^{2}-0.84059x_{2}^{2}-0.29579x_{3}^{2}-0.024965x_{4}^{2}-87.73641 \end{array}$$

(7) $$\begin{array}{l} y_{{\text{k}}_{\text{m}}(\times 10^{6})}=0.015553x_{1}+1.45125x_{2}+1.26430x_{3}+1.25892x_{4}+6.66641\times 10^{-4}x_{1}x_{2}-2.14507\times 10^{-4}x_{1}x_{3}\\ \;-\;5.04257\times 10^{-4}x_{1}x_{4}-2.57349\times 10^{-3}x_{2}x_{3}+5.20183\times 10^{-3}x_{2}x_{4}-0.015768x_{3}x_{4}\\ \;-\;1.37062\times 10^{-6}x_{1}^{2}-0.11317x_{2}^{2}-0.026007x_{3}^{2}-0.013908x_{4}^{2}-31.21950 \end{array}$$

(8) $$\begin{array}{l} y_{\alpha }=-4.82555\times 10^{-3}x_{1}+1.15881x_{2}-2.00075x_{3}+0.68115x_{4}-6.99771\times 10^{-4}x_{1}x_{2}+9.13947\\ \;\times \;10^{-5}x_{1}x_{3}+2.71615\times 10^{-4}x_{1}x_{4}+1.01179\times 10^{-3}x_{2}x_{3}-4.05986\times 10^{-3}x_{2}x_{4}\\ \;-\;1.19447\times 10^{-5}x_{1}^{2}-0.056538x_{2}^{2}+0.081820x_{3}^{2}-0.010587x_{4}^{2}-1.17525 \end{array}$$

(9) $$\begin{array}{l} y_{\text{EC}}=-1.25669x_{1}+38.36969x_{2}+0.45101x_{3}-22.70344x_{4}-0.065281x_{1}x_{2}+0.013980x_{1}x_{3}\\ \;+\;7.91250\times 10^{-3}x_{1}x_{4}-1.75525x_{2}x_{3}-0.32888x_{2}x_{4}+0.10520x_{3}x_{4}+1.50082\times 10^{-3}x_{1}^{2}+2.90536x_{2}^{2}\\ \;-\;0.10089x_{3}^{2}+0.28896x_{4}^{2}+571.36412 \end{array}$$

**Table 3:** ANOVA results of the quadratic models of COD removal, mass transfer coefficient (k_m_), ${\text{J}/\text{J}}_{\text{lim}}^{{}^{\circ}}$ (α) value and energy consumption for the electrochemical oxidation of resorcinol in aqueous medium using BDD anode.

**Source**	**Sum of squares**	**Degrees of freedom**	**Mean square**	***F*-value**	***P*-value**
**COD REMOVAL (s/*****n*** = **6.775)**					
Model	5327.13	14	380.51	2.44	0.0278
Residual	3581.35	23	155.71		
Lack of fit	2772.10	18	154.01	0.95	0.5811
Pure error	809.25	5	161.85		
**MASS TRANSFER COEFFICIENT (s/*****n*** = **5.870)**					
Model	55.22	14	3.94	3.08	0.0082
Residual	29.45	23	1.28		
Lack of fit	24.54	18	1.36	1.39	0.3837
Pure error	4.91	5	0.98		
**${\text{J}/\text{J}}_{\text{lim}}^{{}^{\circ}}$** **(**α**) VALUE (s/*****n*** = **7.952)**					
Model	225.66	13	17.36	2.35	0.0339
Residual	177.41	24	7.39		
Lack of fit	176.91	19	9.31	92.01	<0.0001
Pure error	0.51	5	0.10		
**ENERGY CONSUMPTION (s/*****n*** = **12.951)**					
Model	1.251 × 10^5^	14	8936.15	10.30	<0.0001
Residual	19948.90	23	867.34		
Lack of fit	19543.76	18	1085.76	13.40	0.0046
Pure error	405.14	5	81.03		

Process variables *x*_1_, *x*_2_, *x*_3_, and *x*_4_ were evaluated for design matrix using Design-Expert^©^ 10 and no aliases were found for the response surface quadratic models. The lack of fit test is valid for this study according to degrees of freedom (df) values where minimum 3 df for lack of fit and 4 df for pure error is recommended for a valid lack of fit test. The variance inflation factors (VIF) measures how much the variance of the model coefficient increases due to the lack of orthogonality in the design. The ideal VIF value is 1.00 and if the design has multilinear constraints multicollinearity exists to a greater degree with VIF > 10. The VIF values were obtained as 1.00 and 1.01 for COD removal, k_m_, ${\text{J}/\text{J}}_{\text{lim}}^{{}^{\circ}}$ values and energy consumption indicating that there was no multicollinearity and the coefficients were very well estimated.

*F*-value is the test for comparing the sources mean square to the residual mean square and *p*-value is the probability of seeing the observed *F*-value for factor effects. Model *F*-values of 2.44, 3.08, 2.35, and 10.30 imply the quadratic models are significant for COD removal, k_m_, ${\text{J}/\text{J}}_{\text{lim}}^{{}^{\circ}}$ values and energy consumption, respectively. *P*-values less than 0.05 indicate the model terms are significant and signal/noise (s/n) ratio >4 is desirable for adequate precision. Significant model terms have a real effect on the response and lack of fit. S/n ratios of 6.775, 5.870, 7.952, and 12.951 imply adequate signals and *P* < 0.05 indicate the significance for the quadratic models that encompasses 95% confidence interval for the design space. In Table 3, the lack of fit *F*-values of 0.95 and 1.39 indicate the insignificance for COD removal and mass transfer coefficient; and *F*-values of 92.01 and 13.40 indicate the significance for ${\text{J}/\text{J}}_{\text{lim}}^{{}^{\circ}}$ values and energy consumption. In this study, residual plots followed a normal distribution, studentized residuals and outlier-t plots indicated good approximations for the fitted quadratic models to the response surfaces (diagnostic plots were not shown).

**Figure 1:**
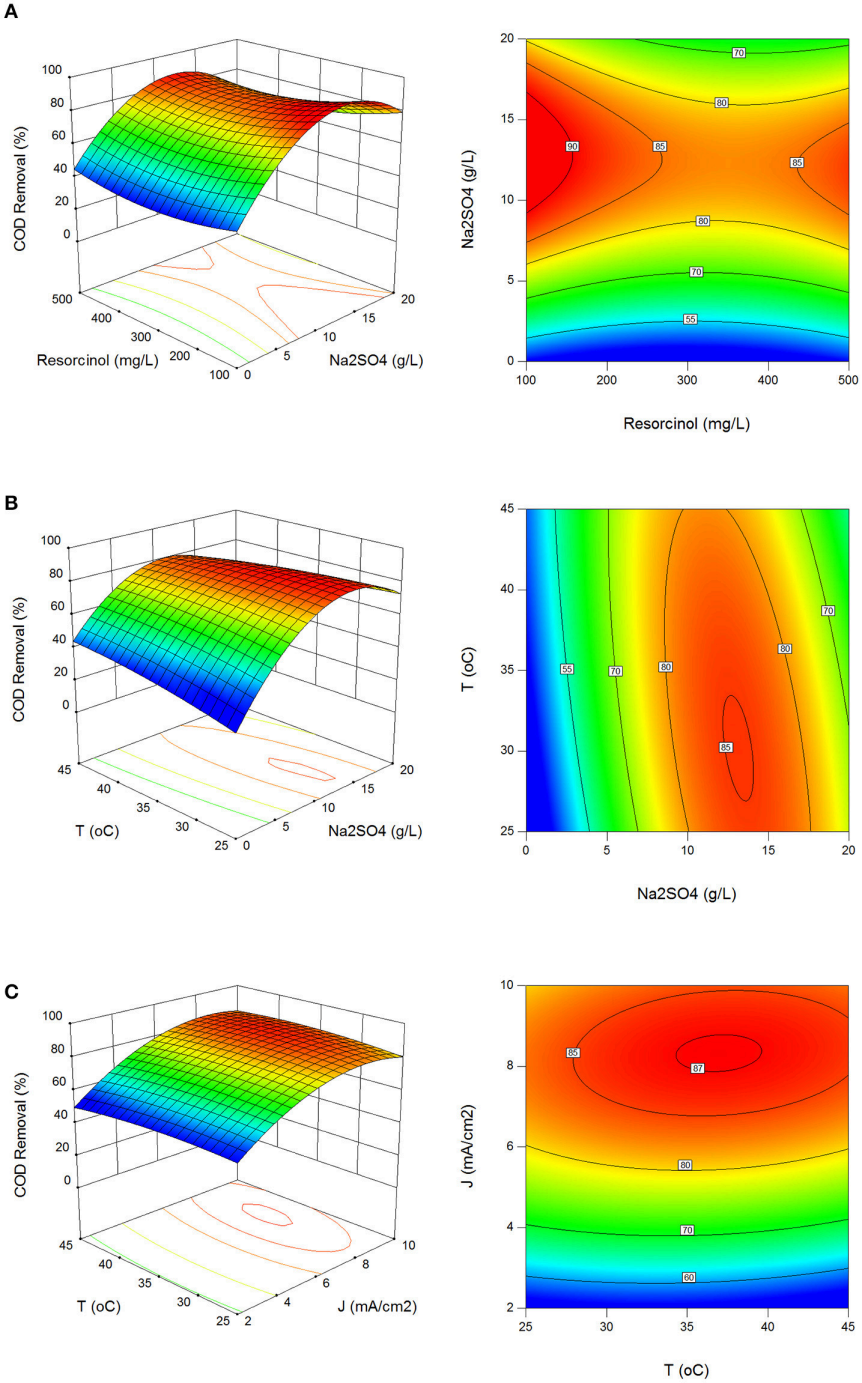
Effect of process parameters on COD removal. **(A)** Effect of resorcinol concentration and Na_2_SO_4_ concentration (*J* = 6 mA/cm^2^, *T* = 35°C, *t* = 120 min), **(B)** effect of Na_2_SO_4_ concentration and reaction temperature (C_o_ = 300 mg/L, *J* = 6 mA/cm^2^, *t* = 120 min), **(C)** effect of reaction temperature and current density (C_o_ = 300 mg/L, Na_2_SO_4_ = 10 g/L, *t* = 120 min).

Figures 1A–C show the effect of process parameters on COD removal in 3D surface and 2D contour plots. COD removal was obtained between 10.6 and 90.3% with standard deviation of 15.5%. Operating region for higher than 85% COD removal was determined at 9.1–14.8 g/L Na_2_SO_4_, 6.8–9.9 mA/cm^2^ current density and 26–34°C reaction temperature in Figures 1A–C. COD decreased by indirect oxidation of resorcinol with the production of redox reagents in the aqueous medium and OH radicals at BDD anode. The production of S_2_${\text{O}}_{8}^{2-}$ oxidizing ions in the presence of Na_2_SO_4_ electrolyte increased the COD removal efficiency as well. Most of the electrochemical processes are based on indirect oxidation because direct oxidation of organic pollutants is very slow at inert anodes due to reaction kinetics, limiting reactions, and mass transport limitations (Rajeshwar and Ibanez, 1997; Körbahti and Artut, 2010; Körbahti and Taşyürek, 2015).

In electrochemical processes, current density is an important parameter for controlling the reaction rate (Körbahti and Taşyürek, 2015). Increasing current density increases the electrochemical oxidation efficiency by increasing the conductivity, and *in situ* production of oxidizing redox reagents in the reaction medium and OH radicals at BDD anode (Körbahti and Taşyürek, 2015). However, gas evolution, electrolyte decomposition and side reactions result in a decrease in the removal yield and a loss of current efficiency during the mineralization of organic pollutants (Fernandes et al., 2004; Shen et al., 2006; Körbahti and Taşyürek, 2015). The electrochemical oxidation of organic compounds including phenols, chlorophenols, cresols, organic acids, naphthol, surfactants, herbicides, cyanides, pharmaceuticals, nitrophenols, polyhydroxybenzenes, polyacrylates, and textile dyes was studied in the literature (Bellagamba et al., 2002; Panizza et al., 2005; Weiss et al., 2007, 2008; Louhichi et al., 2008; González et al., 2011; Körbahti and Taşyürek, 2015), and it was reported that increasing current density increases the removal of organic pollutants, COD removal, and energy consumption.

In direct or indirect electrochemical oxidation side products and reaction intermediates generate before complete mineralization. This behavior was reported for the oxidation of organic compounds on BDD anodes that the oxidation occurs directly on the anode surface or indirectly in the aqueous medium very close to the anode surface mediated by hydroxyl radicals generated by the water oxidation (Cañizares et al., 2003, 2004; Nasr et al., 2005). The amount and the nature of the intermediates depend on the properties of the anodes and the process conditions (Comninellis and Chen, 2010). The main intermediates using BDD anodes were reported as maleic, oxalic, and formic acid which further oxidized at lower rates (Comninellis and Chen, 2010; Martínez-Huitle and Andrade, 2011; Körbahti and Taşyürek, 2015).

Comninellis and Chen (2010) reported that the mineralization reaction rate is independent of the organic compound in the electrolyte. Therefore, mass transfer coefficient could be expressed in electrochemical reactors with the physical parameters of the system such as current density, electrode potential, electrochemical cell voltage, electrode length, electrolysis time, electrode area/electrolyte volume ratio, and electrolyte properties (Pletcher and Walsh, 1990; Rajeshwar and Ibanez, 1997). In Equation (10), mass transfer coefficient for stirred batch electrochemical reactor was expressed using mass balance over the reactor by Faraday's Law under mass transport control with convective diffusion (Pletcher and Walsh, 1990; Rajeshwar and Ibanez, 1997; Körbahti and Artut, 2010; Körbahti and Taşyürek, 2015).

(10) $$x_{SBER}=1-\exp\left(-\frac{k_{m}A_{e}}{V_{R}}t\right)$$

**Figure 2:**
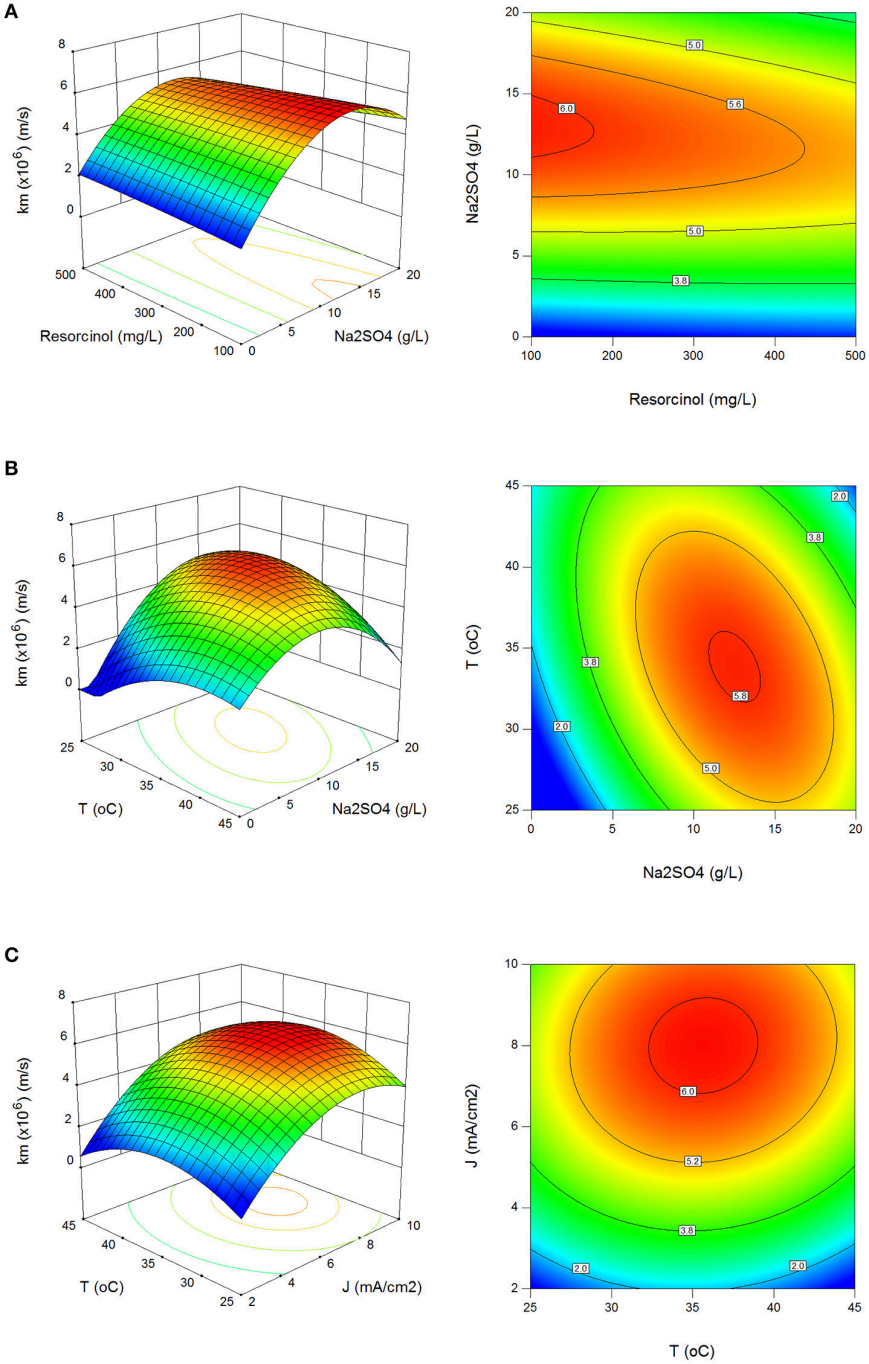
Effect of process parameters on mass transfer coefficient (k_m_). **(A)** Effect of resorcinol concentration and Na_2_SO_4_ concentration (*J* = 6 mA/cm^2^, *T* = 35°C, *t* = 120 min), **(B)** effect of Na_2_SO_4_ concentration and reaction temperature (C_o_ = 300 mg/L, *J* = 6 mA/cm^2^, *t* = 120 min), **(C)** effect of reaction temperature and current density (C_o_ = 300 mg/L, Na_2_SO_4_ = 10 g/L, *t* = 120 min).

The effect of process parameters on mass transfer coefficient (k_m_) in surface and contour plots can be seen in Figures 2A–C. Mass transfer coefficients were evaluated between 0.33 × 10^−6^ and 6.93 × 10^−6^ m/s with standard deviation of 1.51 × 10^−6^ m/s which are consistent to those reported in the literature (Körbahti and Artut, 2010; Körbahti and Taşyürek, 2015). Higher than 5.5 × 10^−6^ m/s mass transfer coefficients were determined in the regions at 5.5–17.8 g/L Na_2_SO_4_, 5.6–10 mA/cm^2^ current density and 28–42°C reaction temperature in Figures 2A–C.

The limiting current density in the beginning of electrolysis (${\text{J}}_{\text{lim}}^{{}^{\circ}}$) can be written under hydrodynamic conditions for the electrochemical mineralization of organic compounds in Equation (11) (Panizza and Cerisola, 2009; Comninellis and Chen, 2010; Martínez-Huitle and Andrade, 2011). The characteristic parameter, α, can be defined for the electrochemical oxidation in Equation (12) (Comninellis and Chen, 2010):

(11) $$J_{lim}^{o}=4Fk_{m}COD_{o}$$

(12) $$\alpha =\frac{J}{J_{lim}^{o}}$$

α is constant under galvanostatic conditions and two different operating conditions can be defined (Panizza and Cerisola, 2009; Comninellis and Chen, 2010; Martínez-Huitle and Andrade, 2011):

1. Electrochemical process is controlled by the applied current, COD decreases linearly with time and current efficiency is 100% (α < 1).
2. Electrochemical process is controlled by the mass transport, secondary reactions involve and COD removal follows an exponential trend due to mass transport limitations results in instantaneous current efficiency (ICE) decrease (α > 1).

**Figure 3:**
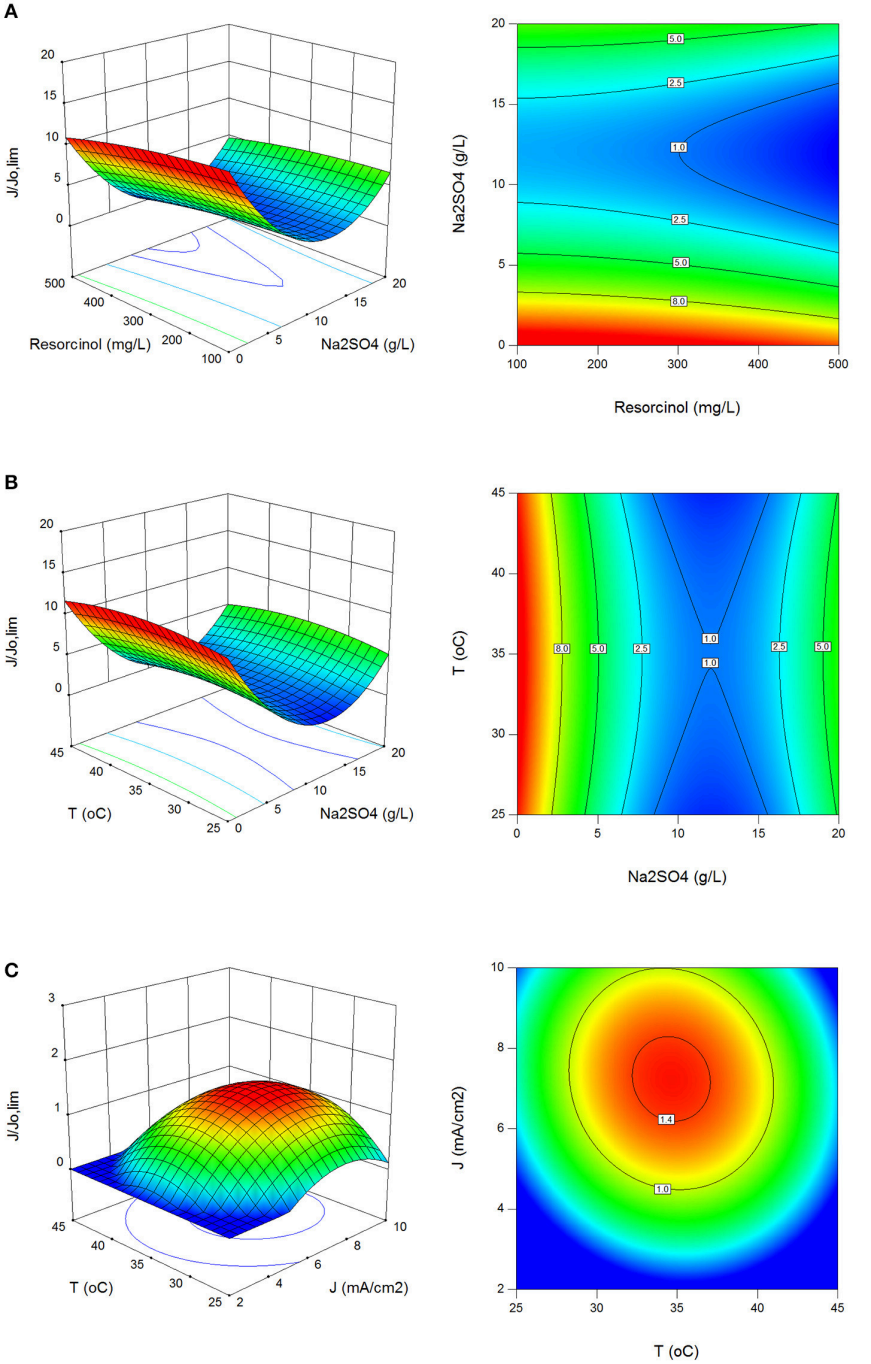
Effect of process parameters on ${\text{J}/\text{J}}_{\text{lim}}^{{}^{\circ}}$ (α). **(A)** Effect of resorcinol concentration and Na_2_SO_4_ concentration (*J* = 6 mA/cm^2^, *T* = 35°C, *t* = 120 min), **(B)** effect of Na_2_SO_4_ concentration and reaction temperature (C_o_ = 300 mg/L, *J* = 6 mA/cm^2^, *t* = 120 min), **(C)** effect of reaction temperature and current density (C_o_ = 300 mg/L, Na_2_SO_4_ = 10 g/L, *t* = 120 min).

In Figures 3A–C the effect of process parameters on ${\text{J}/\text{J}}_{\text{lim}}^{{}^{\circ}}$ (α) values in surface and contour plots is shown. ${\text{J}/\text{J}}_{\text{lim}}^{{}^{\circ}}$ values were obtained between 0.8 and 21.7 with standard deviation of 3.3. Electrolysis under current limited control results in the formation of many intermediates according to the electrolysis under mass transport control (Panizza and Cerisola, 2009; Comninellis and Chen, 2010; Martínez-Huitle and Andrade, 2011). Therefore, the process parameters were optimized for mass transport control in this study. It was found that the electrochemical oxidation of resorcinol using BDD anode should be operated for mass transport control region at initial resorcinol concentration ≤300 mg/L, between 28 and 42°C reaction temperature and 4.5–10 mA/cm^2^ current density.

In electrolysis, electrical charge involves for the chemical changes in non-spontaneous oxidation and reduction reactions (ΔG > 0) and in most cases supplied electrical energy is larger than the Gibbs free energy change of the reactions. Therefore, energy consumption in electrochemical processes should be optimized for energy efficient processes. In the batch runs, energy consumption values were evaluated between 39.0 and 302.7 kWh/kg COD_r_ with standard deviation of 62.6 kWh/kg COD_r_ using Equation (13).

(13) $$EC=\frac{iV_{m}\Delta t}{\left(COD_{O}-COD_{t}\right)V_{R}}$$

**Figure 4:**
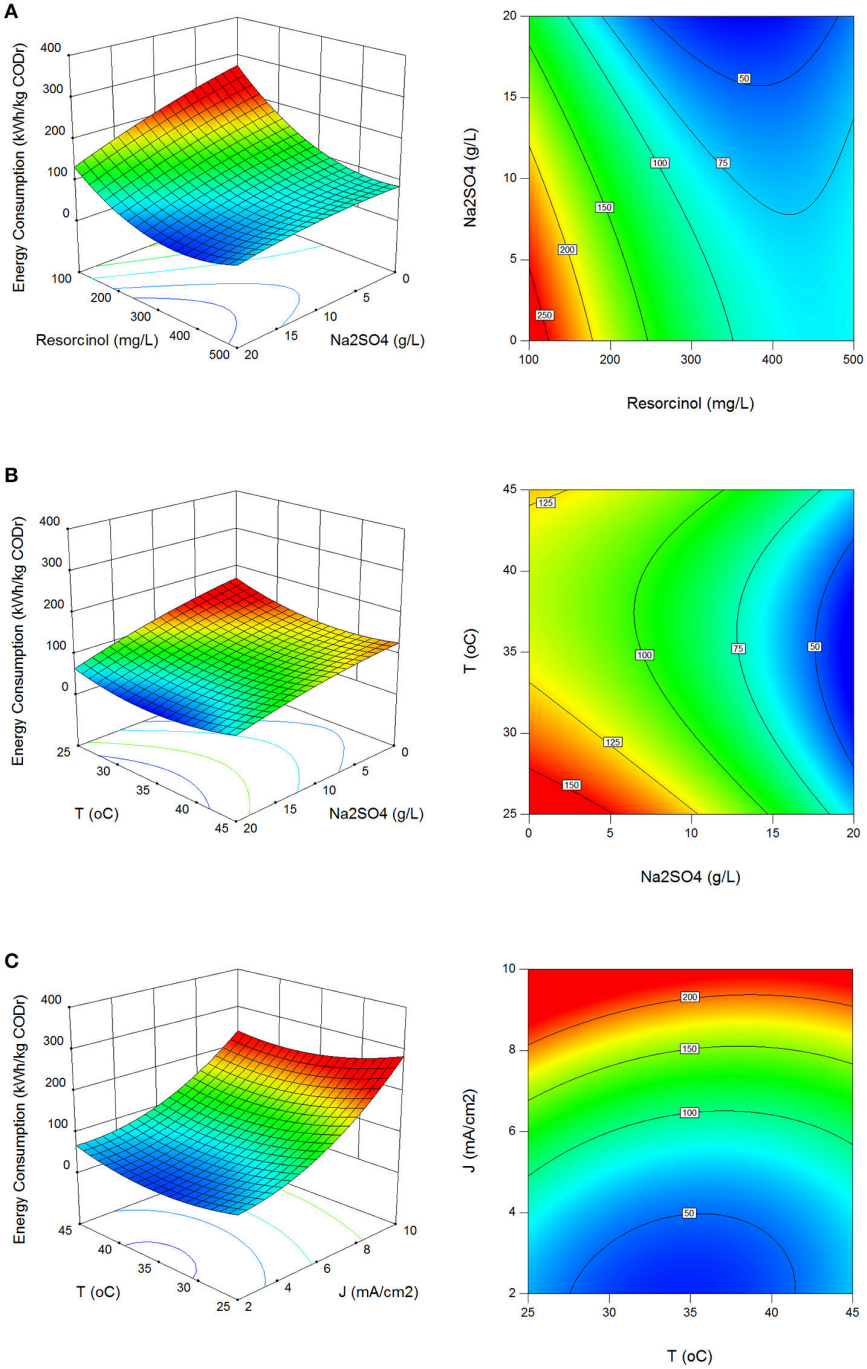
Effect of process parameters on energy consumption. **(A)** Effect of resorcinol concentration and Na_2_SO_4_ concentration (*J* = 6 mA/cm^2^, *T* = 35°C, *t* = 120 min), **(B)** effect of Na_2_SO_4_ concentration and reaction temperature (C_o_ = 300 mg/L, *J* = 6 mA/cm^2^, *t* = 120 min), **(C)** effect of reaction temperature and current density (C_o_ = 300 mg/L, Na_2_SO_4_ = 10 g/L, *t* = 120 min).

Figures 4A–C show the effect of process parameters on energy consumption in surface and contour plots. Energy consumption decreased with the increase in resorcinol concentration and Na_2_SO_4_ concentration, and the decrease in the current density. The energy efficient reaction temperature was found between 28 and 42°C.

**Figure 5:**
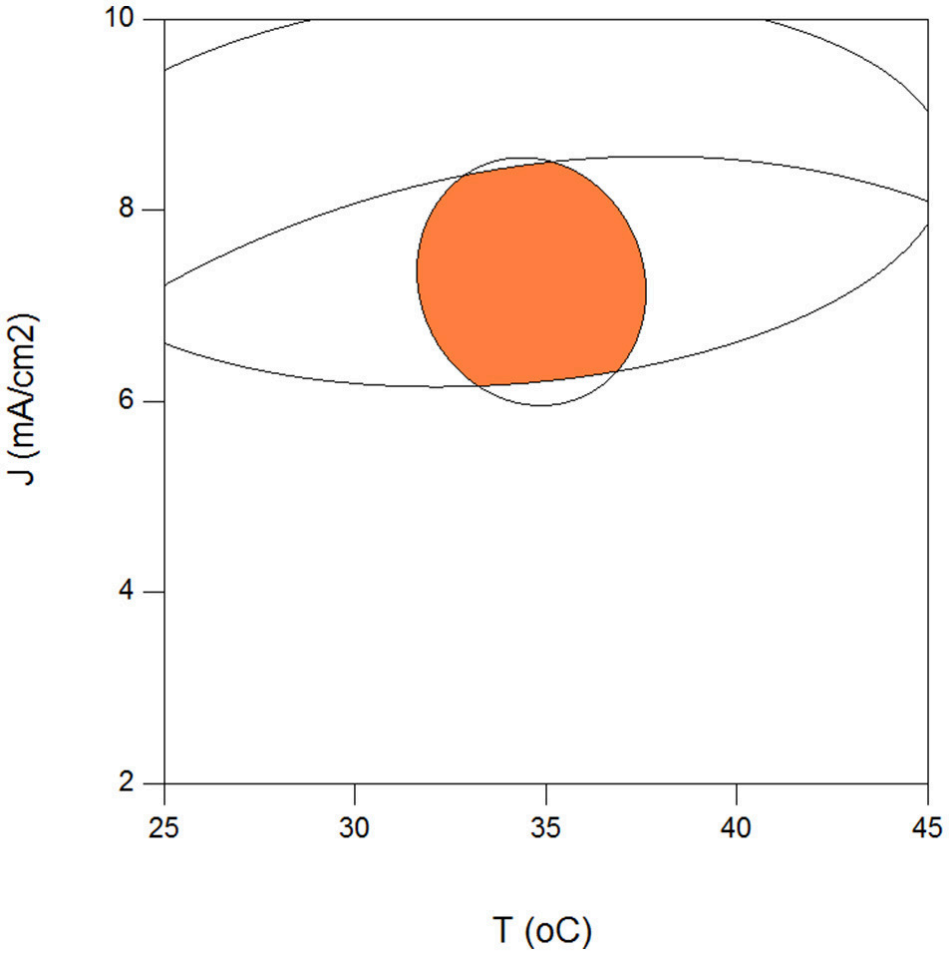
Optimum operating region for the highest electrochemical oxidation efficiency of resorcinol in aqueous medium using BDD anode for COD removal >85%, ${\text{J}/\text{J}}_{\text{lim}}^{{}^{\circ}}$ > 1, energy consumption <150 kWh/kg COD removed (C_o_ = 300 mg/L, COD_o_ = 620 mg/L, Na_2_SO_4_ = 12 g/L, *t* = 120 min).

**Figure 6:**
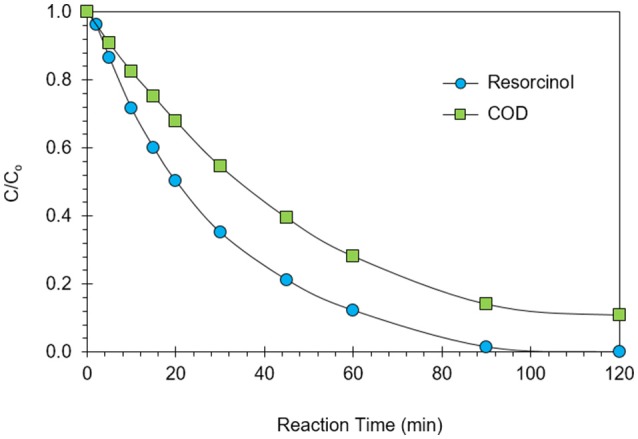
Results of the batch run at response surface optimized conditions for the electrochemical oxidation of resorcinol in aqueous medium using BDD anode (C_o_ = 300 mg/L, COD_o_ = 620 mg/L, *J* = 8 mA/cm^2^, Na_2_SO_4_ = 12 g/L, *T* = 34°C, *t* = 120 min).

Maximization of COD removal efficiency was preferred for the process optimization, and optimum process parameters were determined as 300 mg/L resorcinol concentration, 8 mA/cm^2^ current density, 12 g/L Na_2_SO_4_ concentration and 34°C reaction temperature by analyzing Figures 1–4. In Figure 5, the shaded region shows the process efficiency for mass transfer controlled process at >85% COD removal and < 150 kWh/kg COD_r_ energy consumption in 95% confidence interval. The specific batch run was conducted at response surface optimized conditions and the results are presented in Figure 6 and Table 4. It can be seen that the relationships developed between the responses and the process parameters in electrochemical oxidation of resorcinol using BDD anode were in very good agreement according to the relative error values lower than ±5% as presented in Table 4. In Figure 6, 100% resorcinol removal and 89% COD removal were obtained in 120 min reaction time. The initial rate of resorcinol oxidation was found higher than the COD removal. In Figure 6, the initial rates were calculated as 0.0257 and 0.0150 min^−1^ for resorcinol oxidation and COD removal, respectively. These results confirmed that the electrochemical mineralization of resorcinol was successfully accomplished using BDD anode depending on the process conditions, however the formation of intermediates and by-products were further oxidized at much lower rate. Brillas and Martínez-Huitle (2015) also reported the formation of by-products such as aliphatic carboxylic acids in electrochemical treatment which are more difficult to oxidize with BDD(^•^OH) than the aromatic pollutants.

**Table 4:** Results of the batch run at response surface optimized conditions for the electrochemical oxidation of resorcinol in aqueous medium using BDD anode (C_o_ = 300 mg/L, COD_o_ = 620 mg/L, *J* = 8 mA/cm^2^, Na_2_SO_4_ = 12 g/L, *T* = 34°C, *t* = 120 min).

**Response**	**Experimental result**	**RSM model**	**Error (±%)**
pH	4.6	4.4	4.3
COD removal (%)	89.0	88.5	0.6
k_m_ (m/s)	6.58 × 10^−6^	6.26 × 10^−6^	4.9
$J_{lim}^{o}$(mA/cm^2^)	4.9	4.7	4.1
$\alpha =\frac{J}{J_{lim}^{o}}$	Mass transfer controlled	Mass transfer controlled	–
Energy consumption (kWh/kg COD_r_)	138.0	134.7	2.4

Nasr et al. (2005) investigated the electrochemical oxidation of hydroquinone, resorcinol and catechol on boron-doped diamond anodes. The authors conducted the research at 13.5 mM catechol, resorcinol or hydroquinone concentration, 3333.33 mg/L Na_3_PO_4_ electrolyte concentration, pH 2, 30 mA/cm^2^ current density and 25°C reaction temperature. Nasr et al. (2005) reported that no aromatic intermediates were found during the treatment and aliphatic intermediates such as carboxylic acids were detected. The authors also observed that the oxidation of resorcinol leads to the formation of important amounts of glycolic and glyoxalic acids. The higher concentrations of C_2_ carboxylic acids indicate their lower oxidazability in comparison with the initial aromatic compounds and with the C_1_ and C_4_ carboxylic acids (Nasr et al., 2005).

On the other hand, it is also possible to obtain mono-, di-, and tri- substituted chlorinated aromatic intermediates during the electrochemical oxidation of phenol in the presence of NaCl electrolyte (Comninellis and Nerini, 1995; Zareie et al., 2001; Körbahti et al., 2002). However, Comninellis and Chen (2010) indicated that electrochemical oxidation could be effectively used for the mineralization of toxic and biorefractory organic pollutants by using diamond film electrodes. Cañizares et al. (2006) also reported the results of the electrolysis at BDD of different substituted phenols showing a significant decrease of the toxicity during the treatment.

The other important issue is the formation of polymer films during the oxidation of dihydroxybenzenes in aqueous medium. It is known that the first stage in the oxidation of dihydroxybenzenes is the formation of a phenoxy radical that can be further oxidized to the quinone form or can couple with other radicals or dihydroxybenzenes to form polymers (Nasr et al., 2005). In our study, the polymer formation did not observe on the BDD surface and in the study of Nasr et al. (2005) as well. The authors indicated that the polymer formed must be easily removed by the hydroxyl radicals formed during the water decomposition (Nasr et al., 2005).

The electrochemical oxidation of resorcinol using BDD anode in aqueous medium was occurred irreversibly in a mass transport controlled process. BDD anode was proved for the electrochemical oxidation of resorcinol with its inert surface, good conductivity, corrosion resistance, and stability. Indirect oxidation was the dominating process as reported in the literature. Resorcinol and the reaction intermediates were oxidized mainly by the hydroxyl radicals produced by water oxidation on the BDD anode surface, and peroxodisulfate produced at high overpotential supplied by the BDD anode in the presence of ${\text{SO}}_{4}^{2-}$ ions in the electrolyte (Serrano et al., 2002; Chen et al., 2004; Cañizares et al., 2005a; Nasr et al., 2005; Panizza and Cerisola, 2007; Comninellis et al., 2008; Weiss et al., 2008; Liu et al., 2009; Li et al., 2010; Sirés and Brillas, 2012; Davis et al., 2014). BDD anode could be used as a favorable electrode material for the total mineralization of organic pollutants that can be found in the wastewater.

### Reaction kinetics for electrochemical oxidation of resorcinol in aqueous medium using BDD anode

Reaction kinetics for electrochemical oxidation of resorcinol in aqueous medium using BDD anode was investigated by the method of initial rates in a batch electrochemical reactor at response surface optimized conditions. The overall electrochemical oxidation rate of pollutants in a batch electrochemical reactor can be expressed based on COD concentration as shown in Equation (14) (Chiang et al., 1997; Lin et al., 1998; Szpyrkowicz et al., 2001a,b, 2005; Körbahti and Artut, 2010).

(14) $$-\frac{dC_{COD}}{dt}=kC_{COD}^{n}$$

(15) $$ln\left(-\frac{dC_{COD}}{dt}\right)=ln\left(k\right)+n.ln\left(C_{COD}\right)$$

**Figure 7:**
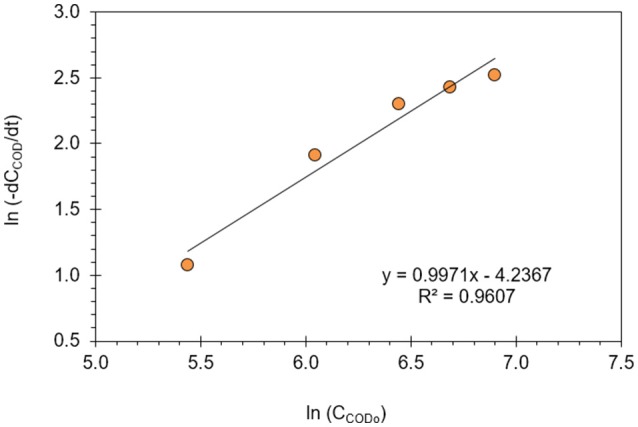
Reaction order (*n*) and reaction rate constant (*k*) of electrochemical oxidation of resorcinol in aqueous medium using BDD anode (*J* = 8 mA/cm^2^, Na_2_SO_4_ = 12 g/L, *T* = 34°C).

Equation (14) can be linearized into Equation (15) for the evaluation of reaction order (*n*) and reaction rate constant (*k*) from batch reactor data. In Figure 7, the reaction order and reaction rate constant at 34°C optimum reaction temperature were determined with linear regression (*R*^2^ = 0.96) as 1 and 0.0145 min^−1^, respectively. Liu et al. (2009) investigated the electrochemical degradation of chlorobenzene on boron-doped diamond and platinum electrodes, and compared the degradation kinetics on these electrodes. The authors reported that the decay of chlorobenzene on BDD and Pt electrodes were both pseudo-first-order reactions, and the reaction rate constant on BDD was calculated as 0.0118 min^−1^ which was higher than on the Pt electrode (Liu et al., 2009). It was also reported in the literature that first-order reaction kinetics for COD removal in the degradation of organic compounds is appropriate for the entire concentration range and several studies were reasonably well fitted to this kinetic model in electrochemical wastewater treatment processes depending on the process conditions (Xiong et al., 2001; Rajkumar and Palanivelu, 2004; Szpyrkowicz et al., 2005; Kong et al., 2006; Panizza and Cerisola, 2006, 2009; Koparal et al., 2007; Brillas et al., 2009; Radha et al., 2009; Körbahti and Artut, 2010; Brillas and Martínez-Huitle, 2015).

The reaction rate constant (*k*) can be defined with Arrhenius equation (Equation 16) and that can be linearized into Equation (17) when overall reaction rate is expressed with Equation (14).

(16) $$k=A.exp\left(-\frac{E_{a}}{RT}\right)$$

(17) $$ln\left(k\right)=ln\left(A\right)-\frac{E_{a}}{RT}$$

**Figure 8:**
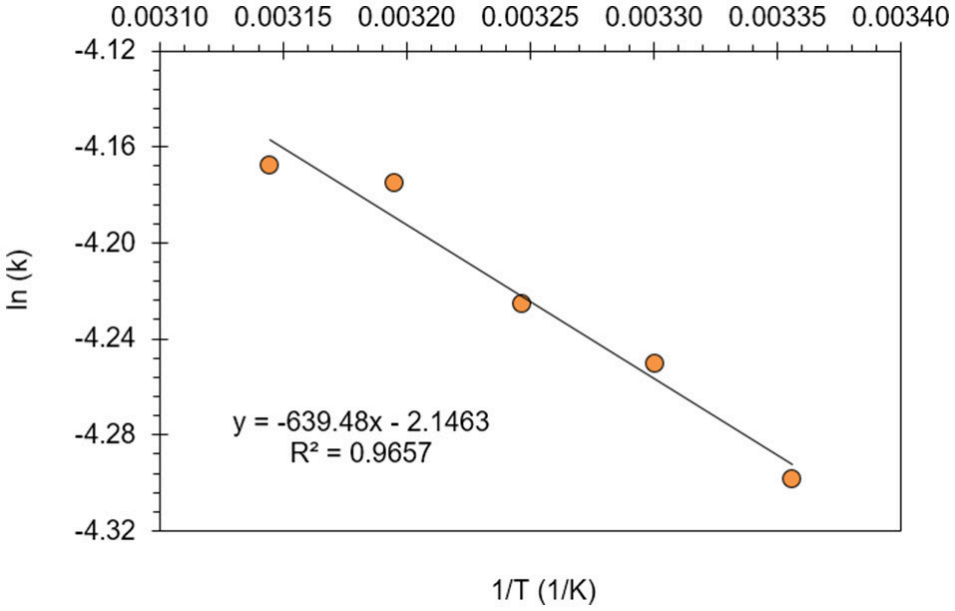
Activation energy (*E*_*a*_) and frequency factor (*A*) determination for the electrochemical oxidation of resorcinol in aqueous medium using BDD anode (C_o_ = 300 mg/L, COD_o_ = 620 mg/L, *J* = 8 mA/cm^2^, Na_2_SO_4_ = 12 g/L).

In Figure 8, the reaction rate constant (*k*) was calculated between 25 and 45°C reaction temperatures and plotted vs. 1/T with linear regression (*R*^2^ = 0.97). The activation energy (*E*_*a*_) and the frequency factor (*A*) were evaluated at 300 mg/L optimum resorcinol concentration as 5.32 kJ/mol and 0.1169 min^−1^, respectively. The activation energy depends on the nature of the reaction and fast reactions generally have small *E*_*a*_ values. It is known that the activation energy for a diffusion-controlled homogeneous reaction is < 40 kJ/mol (Samet et al., 2006; Körbahti and Artut, 2010). Therefore, the electrochemical oxidation of resorcinol in aqueous medium using BDD anode was supported a diffusion-controlled reaction.

## Concluding remarks

In this study, the electrochemical oxidation of resorcinol in aqueous medium using boron-doped diamond (BDD) anode was investigated in a batch electrochemical reactor. The following conclusions were drawn from the study:

- The effect of process parameters on COD removal, mass transfer coefficient (k_m_), ${\text{J}/\text{J}}_{\text{lim}}^{{}^{\circ}}$ values (α), and energy consumption were evaluated. Depending on the process conditions.
  - COD removal was obtained between 10.6 and 90.3% in 120 min reaction time.
  - Mass transfer coefficients (k_m_) were calculated between 0.33 × 10^−6^ and 6.93 × 10^−6^ m/s.
  - Energy consumption values were evaluated between 39.0 and 302.7 kWh/kg COD_r_.
  - ${\text{J}/\text{J}}_{\text{lim}}^{{}^{\circ}}$ values (α) were calculated between 0.8 and 21.7 and the process was optimized for the operation in mass transport control region (α > 1) using RSM.
- Optimum process parameters were determined as C_o_ = 300 mg/L, *J* = 8 mA/cm^2^, Na_2_SO_4_ = 12 g/L, *T* = 34°C for mass transfer controlled process at >85% COD removal and <150 kWh/kg COD_r_ energy consumption.
- One hundred percent of resorcinol removal and 89% COD removal were obtained at response surface optimized conditions in 120 min reaction time.
- Initial rate of resorcinol oxidation was found higher than the COD removal.
- Electrochemical oxidation of resorcinol in aqueous medium using BDD anode was determined as first order reaction based on COD concentration with the activation energy of 5.32 kJ/mol that was supported a diffusion-controlled reaction.
- The relationships developed between the responses and the process parameters were in very good agreement according to the relative error values < ±5%.

The experimental results of this study showed the feasibility of using boron-doped diamond (BDD) anode in electrochemical oxidation of phenolic effluents that can be found in domestic and industrial wastewater.

## Author contributions

PD conducted the literature review, performed the experiments, analyzed the results, and prepared the draft manuscript. BK guided in design of experiments, response surface methodology, process optimization and reaction kinetics, and revised the manuscript. BK is the supervisor of this research.

### Conflict of interest statement

The authors declare that the research was conducted in the absence of any commercial or financial relationships that could be construed as a potential conflict of interest.

## Abbreviations

A: frequency factor (min^−1^)
A_e_: electrode area (m^2^)
ANOVA: analysis of variance
BDD: boron-doped diamond
C_o_: initial resorcinol concentration (mg/L)
CCD: central composite design
COD: chemical oxygen demand (mg/L)
COD_o_: initial chemical oxygen demand at *t* = 0 (mg/L)
COD_f_: final chemical oxygen demand at *t* = 120 min (mg/L)
COD_r_: COD removed
E_a_: activation energy (kJ/mol)
EC: energy consumption (kWh/kg COD_r_)
F: Faraday constant (96,485 C/mol)
J: current density (mA/cm^2^)
${\text{J}}_{\text{lim}}^{\text{o}}$: initial limiting current density (mA/cm^2^)
k: number of process parameters (factors) in Equation (5)
k: reaction rate constant (min^−1^) in Equations (14–17)
k_m_: mass transfer coefficient (m/s)
n: reaction order
RSM: response surface methodology
t: reaction time (min)
T: reaction temperature (°C)
V_m_: mean cell voltage (Volt)
V_R_: reaction volume (mL)
*x*_i_, *x*_j_: process parameters (factors)
*x*_*SBER*_: fractional COD conversion in stirred batch electrochemical reactor
*y*: predicted value of the model (response); **Greek Letters**
α: parameter determines the location of the points in experimental design in Table 1
α: ${\text{J}/\text{J}}_{\text{lim}}^{\text{o}}$ ratio in Equation (12)
β_o_, β_i_, β_ii_, β_ij_: constant, linear, quadratic and interaction coefficients in the model
Δt: reaction time (min).
